# EMT Features in Claudin-Low versus Claudin-Non-Suppressed Breast Cancers and the Role of Epigenetic Modifications

**DOI:** 10.3390/cimb45070381

**Published:** 2023-07-19

**Authors:** Ioannis A. Voutsadakis

**Affiliations:** 1 Algoma District Cancer Program, Sault Area Hospital, 750 Great Northern Road, Sault Ste Marie, ON P6B 0A8, Canada; ivoutsadakis@yahoo.com or ivoutsadakis@nosm.ca; 2 Section of Internal Medicine, Division of Clinical Sciences, Northern Ontario School of Medicine, Sudbury, ON P3E 2C6, Canada

**Keywords:** claudins, epigenetic, epithelial-to-mesenchymal transition, plasticity, methylation

## Abstract

Background: Breast cancers are heterogeneous and are classified according to the expression of ER, PR and HER2 receptors to distinct groups with prognostic and therapeutic implications. Within the triple-negative group, with no expression of these three receptors, molecular heterogeneity exists but is currently not exploited in the clinic. The claudin-low phenotype is present in a subset of triple-negative breast cancers and constitutes together with basal-like cancers the most extensive groups within triple-negative breast cancers. Suppression of epithelial cell adhesion molecules in claudin-low cancers is also a hallmark of Epithelial Mesenchymal Transition (EMT). Methods: The groups of claudin-low and claudin-non-suppressed breast cancers from the extensive publicly available genomic cohorts of the METABRIC study were examined to delineate and compare their molecular landscape. Genetic and epigenetic alterations of key factors involved in EMT and potentially associated with the pathogenesis of the claudin-low phenotype were analyzed in the two groups. Results: Claudin-low cancers displayed up-regulation of several core transcription factors of EMT at the mRNA level, compared with claudin-non-suppressed breast cancers. Global promoter DNA methylation was increased in both groups of triple-negative cancers and in claudin-low ER-positive cancers compared with the rest of ER-positive cancers. Histone modifier enzymes, including methyltransferases, demethylases, acetyltransferases and deacetylases displayed amplifications more frequently in claudin-non-suppressed triple-negative cancers than in claudin-low counterparts and the expression of some of these enzymes differed significantly between the two groups. Conclusion: Claudin-low and claudin-non-suppressed triple-negative breast cancers differ in their landscape of EMT core regulators and epigenetic regulators. These differences may be explored as targets for therapeutic interventions specific to the two groups of triple-negative breast cancers.

## 1. Introduction

Breast cancer is the most prevalent female cancer and a significant cause of morbidity and mortality in the United States and globally [1,2]. It is a heterogeneous disease with most cases expressing the hormone nuclear receptors Estrogen Receptor (ER) and Progesterone Receptor (PR). A subset of breast cancers expresses, instead or in addition to the hormone receptors, the receptor of the Epidermal Growth Factor Receptor family HER2. However, about 15% of all breast cancers called triple-negative express neither hormone receptors nor HER2 [3]. This group is characterized by a more aggressive behavior and a higher propensity for recurrence and metastases. Immunohistochemical and genomic studies have disclosed further heterogeneity within the triple-negative group [4,5,6]. Genomic classifications of triple-negative breast cancers performed by various groups of investigators identified diverse triple-negative breast cancer subtypes that are peculiar to each classification, but general similarities are evident [6,7,8]. Two main clusters are identified in all classifications, a basal-like cluster, which is sub-divided, depending on immune-related characteristics, in an immune-activated and an immune-suppressed group, and a mesenchymal/claudin-low cluster. A third cluster observed in all genomic classifications consists of luminal-like triple-negative cancers that express, instead of ER and PR, the Androgen Receptor (AR) [5,6,7,8].

The claudin-low group of breast cancers presents, as a defining characteristic, the down-regulation of the expression of adhesion proteins such as claudins 3, 4 and 7, occludin and E cadherin. These cancers are mostly, but not exclusively, triple-negative by immunohistochemistry [9,10]. The down-regulation of adhesion molecules results in the loss of epithelial cell adhesions and the up-regulation of mesenchymal proteins such as vimentin and N cadherin [11]. Together with the loss of adhesion molecules and expression of mesenchymal proteins, claudin-low cancers acquire features of the epithelial mesenchymal transition (EMT), a program operational in embryogenesis that is instrumental during organogenesis and fetal development [12]. EMT as observed in cancer is a continuum of states between the epithelial and mesenchymal state and subsets of cancer cells are found in different points along the continuum in a given time [13]. Cancer cells transitioning across the continuum are also versatile in reversing back to an epithelial state; thus, EMT and the reverse process, mesenchymal epithelial transition (MET) are together referred to as epithelial mesenchymal plasticity (EMP). Plasticity acquired through the EMT programs is associated with stem cell characteristics and increased renewal capabilities [14,15].

Claudin-low cancers are characterized by low levels of genomic instability [16]. Thus, EMT properties associated with claudin-low breast cancers are acquired without extensive genetic alterations in their genomes. In contrast, reversible epigenetic and post-transcriptional regulations are instrumental in the plasticity observed during the execution of EMP programs [17]. In the current investigation, claudin-low breast cancers are compared with cancers without claudin suppression, with a focus on EMT regulations, to identify molecular alterations that define these subtypes and contribute to their molecular pathogenesis.

## 2. Methods

### 2.1. Genomic Studies and Genomic Classifications

Most of the analyses reported in the current investigation were based on the METABRIC (Molecular Taxonomy of Breast Cancer International Consortium) study cohort and the breast cancer cohort of The Cancer Genome Atlas (TCGA) [10,18,19]. The METABRIC genomic study of breast cancer includes over 2400 breast cancer patient samples analyzed with a targeted next-generation sequencing panel [10]. For analyses of genes that were not included in the targeted panel used in the METABRIC, additional insights were obtained by the evaluation of the breast cancer cohort of TCGA, which had used a whole exome approach [19]. Both the METABRIC and TCGA cohorts are annotated in the cBioportal for cancer genomics site (http://www.cbioportal.org, accessed on 29 May 2023), where all analyses were performed [20,21]. The cBioPortal is a genomics site developed by investigators at the Memorial Sloan Kettering Cancer Center (MSKCC) and currently maintained by MSKCC in collaboration with other investigators, and allows for the interrogation of various genomic studies included in its database for the identification of molecular alterations of interest at the individual patient level, in an anonymized manner [20,21].

The METABRIC study categorizes participating breast cancer patients according to both a clinical classification based on ER, PR, HER2 and proliferation marker Ki67 expressions, as used clinically and also according to the genomic PAM50 (Prediction Analysis of Microarray 50) plus claudin-low classification. This latter genomic classification encompasses the classic categories luminal A, luminal B, HER2-enriched and basal-like as proposed initially by Perou et al., and later added a claudin-low group [22,23,24]. Copy number alterations were determined in METABRIC using the GISTIC2 (Genomic Identification of Significant Targets in Cancer) algorithm, which considers a gene as putatively amplified if it has a score of 2 or above, while genes with a score of −2 or below were considered deleted [25]. For the mRNA expression normalization, the RSEM (RNA Sequencing by Expectation-Maximization) algorithm was used [26].

### 2.2. Statistical Analysis

Statistical comparisons of categorical data were performed with the Fisher’s exact test and the x^2^ test and comparisons of continuous data were performed with the *t* test. The Bonferroni procedure was used for the correction for multiple comparisons. All statistical comparisons were considered significant if *p* < 0.05.

## 3. Results

**Figure 1:**
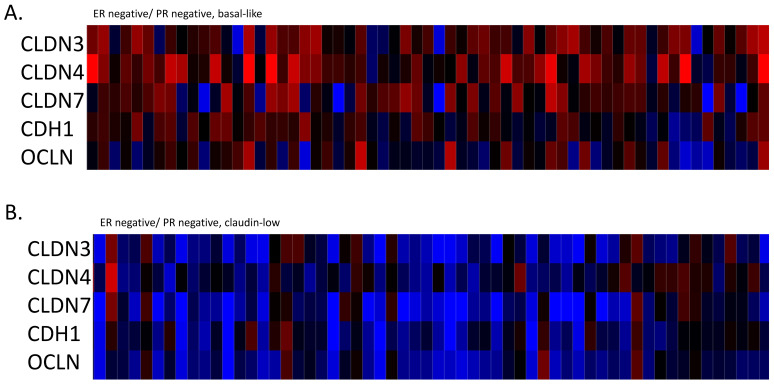

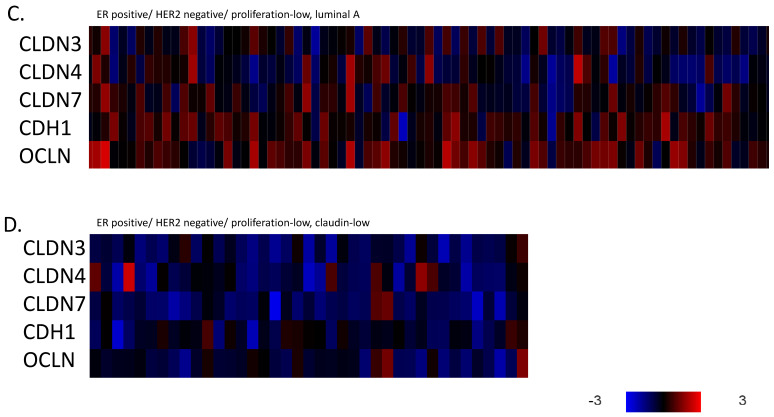
mRNA expression calculated as z-scores relative to all samples (log RNA Seq RPKM) of claudins 3, 4 and 7, E cadherin and occludin in representative breast cancer cases. (**A**). ER-negative/PR-negative, basal-like cancers, (**B**). ER-negative/PR-negative, claudin-low cancers, (**C**). ER-positive/HER2-negative/proliferation-low, luminal A cancers, (**D**). ER-positive/HER2-negative/proliferation-low, claudin-low cancers. Data are from the METABRIC cohort. Red color denotes up-regulation and blue denotes suppression. Gene symbols *CLDN3*: Claudin 3, *CLDN4*: Claudin 4, *CLDN7*: Claudin 7, *CDH1*: E cadherin, *OCLN*: Occludin.

A subset of ER-negative/HER2-negative breast cancers in the METABRIC cohort had the claudin-low phenotype. These cases, representing about 40% of ER-negative/HER2-negative breast cancers in this cohort, displayed down-regulated mRNA expression of adhesion molecules claudin 3, claudin 4, claudin 7, E cadherin and occludin compared with basal ER-negative/HER2-negative breast cancers and luminal A, ER-positive/HER2-negative breast cancers (Figure 1A–C). A smaller subset of 6% of ER-positive/HER2-negative breast cancers with a low proliferation index also displayed the claudin-low phenotype and a suppressed claudin mRNA expression (Figure 1D). This down-regulation was due to transcriptional or post-transcriptional deregulation, given that these cancers did not possess deep deletions or mutations in claudin genes, *CLDN3*, *CLDN4* or *CLDN7*, and only 5% of claudin-low cases had deep deletions in the occludin gene *OCLN*.

**Figure 2:**
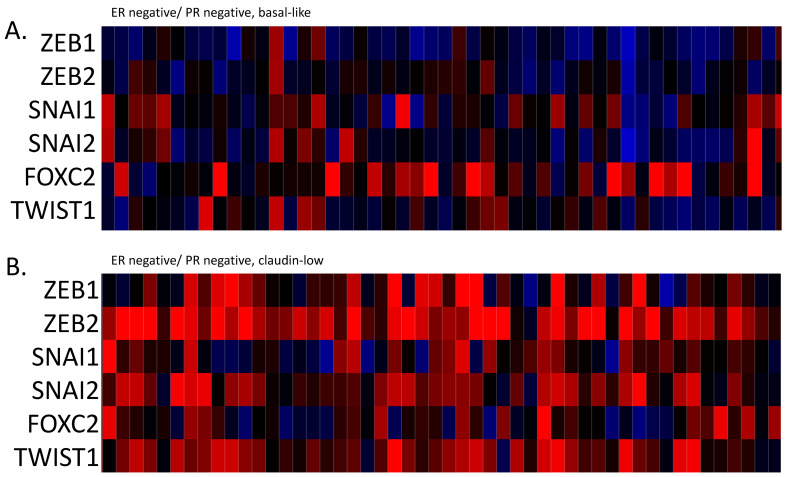

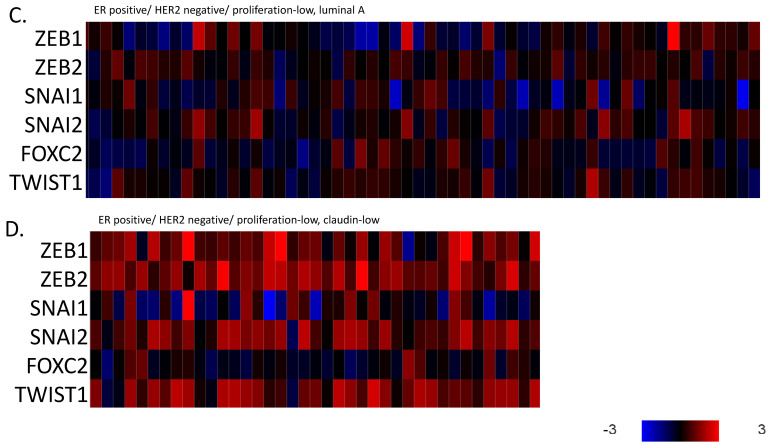
mRNA expression calculated as z-scores relative to all samples (log RNA Seq RPKM) of transcription regulators ZEB1, ZEB2, SNAIL (Gene symbol: SNAI1), Slug (Gene symbol: SNAI2), FOXC2 and TWIST1 in representative breast cancer cases. (**A**). ER-negative/PR-negative, basal-like cancers, (**B**). ER-negative/PR-negative, claudin-low cancers, (**C**). ER-positive/HER2-negative/proliferation-low, luminal A cancers, (**D**). ER-positive/HER2-negative/proliferation-low, claudin-low cancers. Data are from the METABRIC cohort. Red color denotes up-regulation and blue denotes suppression.

**Figure 3:**
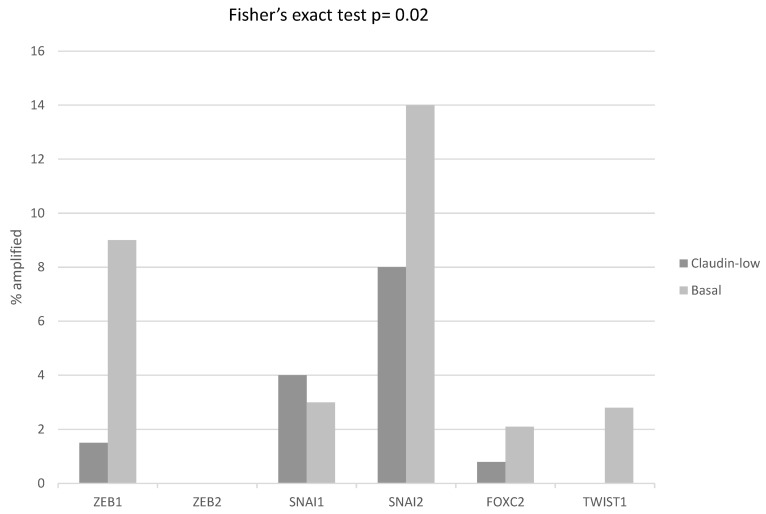
Percentage of amplifications of transcription regulators ZEB1, ZEB2, SNAIL (Gene symbol: *SNAI1*), Slug (Gene symbol: *SNAI2*), FOXC2 and TWIST1 in ER-negative/PR-negative, basal-like breast cancers (grey bars) and ER-negative/PR-negative, claudin-low breast cancers (black bars). Data are from the METABRIC cohort. Fisher’s exact test *p* = 0.02 for the comparison of the presence of amplifications in any core EMT transcription factor in claudin-low versus basal-like breast cancers.

The mRNA expression of core EMT transcription factors ZEB1, ZEB2, SNAI1, SNAI2, and TWIST1 displayed a reverse correlation with claudin expression, and was up-regulated in claudin-low breast cancers, while basal ER-negative/HER2-negative and ER-positive/HER2-negative cancers with no claudin suppression displayed lower levels of expression of these core EMT transcription regulators (Figure 2). FOXC2, also considered an EMT related factor, did not show this correlation with the claudin-low phenotype, as it was less consistently elevated in claudin-low cases and it showed up-regulation in several basal-like cases (Figure 2). Similar to the regulation of claudins, the up-regulation of core transcription factors in claudin-low breast cancers was at the transcriptional or post-transcriptional level, as only a small percentage of cases displayed amplifications in the respective genes, which in fact was higher in basal ER-negative/HER2-negative cancers (Figure 3). Amplification in one or more EMT core regulators was present in 18 of 130 (13.8%) claudin-low ER-negative/HER2-negative cases and in 36 of 143 (25.2%) basal ER-negative/HER2-negative cases (Fisher’s exact test *p* = 0.02). Mutation data for the core EMT regulators were not provided in the METABRIC series. However, data from the breast cancer cohort of TCGA, which did not differentiate between basal and claudin-low cases in cBioportal, confirmed that these mutations are rare.

**Figure 4:**
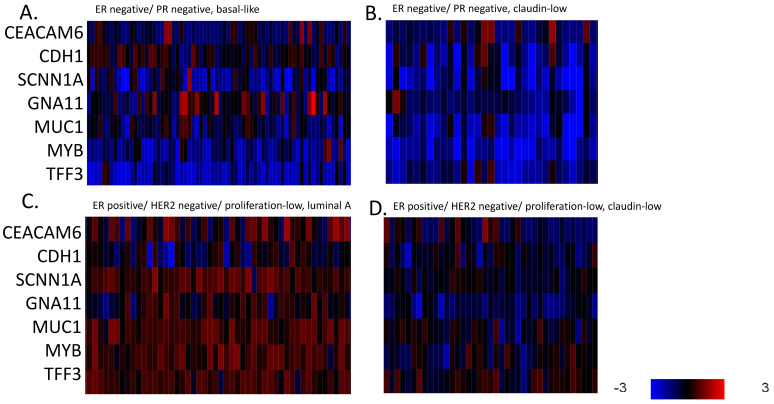
mRNA expression calculated as z-scores relative to all samples (log RNA Seq RPKM) of methylation-sensitive genes in representative breast cancer cases. (**A**). ER-negative/PR-negative, basal-like cancers, (**B**). ER-negative/PR-negative, claudin-low cancers, (**C**). ER-positive/HER2-negative/proliferation-low, luminal A cancers, (**D**). ER-positive/HER2-negative/proliferation-low, claudin-low cancers. Data are from the METABRIC cohort. Red color denotes up-regulation and blue denotes suppression.

**Figure 5:**
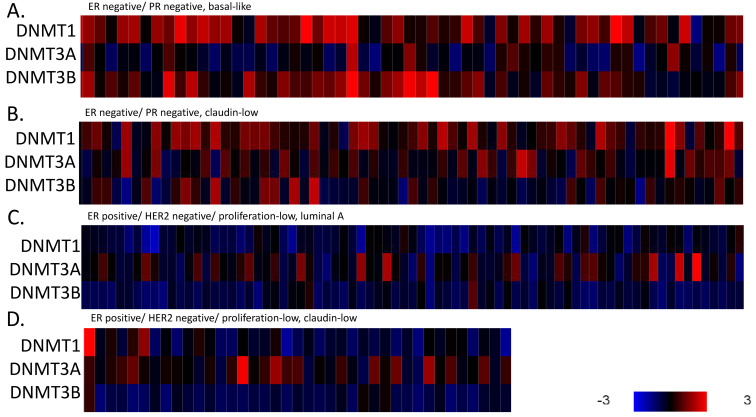
mRNA expression calculated as z-scores relative to all samples (log RNA Seq RPKM) of DNA methyltransferases DNMT1, DNMT3A and DNMT3B in representative breast cancer cases. (**A**). ER-negative/PR-negative, basal-like cancers, (**B**). ER-negative/PR-negative, claudin-low cancers, (**C**). ER-positive/HER2-negative/proliferation-low, luminal A cancers, (**D**). ER-positive/HER2-negative/proliferation-low, claudin-low cancers. Data are from the METABRIC cohort. Red color denotes up-regulation and blue denotes suppression.

Epigenetic regulations, including DNA methylation and histone methylation and acetylation, play a major role in transcription regulation and are an integral part of the molecular landscape of the claudin-low phenotype. DNA methylation-sensitive genes (a panel consisting of *CEACAM6*, *CDH1*, *SCNN1A*, *GNA11*, *MUC1*, *MYB* and *TFF3*) were down-regulated in both basal and claudin-low ER-negative/HER2-negative cases, while claudin-non-suppressed ER-positive/HER2-negative cases and, to a lesser degree, claudin-low ER-positive/HER2-negative cases showed increased expression of the methylation-sensitive gene set (Figure 4). These results suggest that hypermethylation is associated with ER negativity rather than exclusively with the claudin-low phenotype. The human methyltransferase enzymes that perform DNA methylation, *DNMT1*, *DNMT3A* and *DNMT3B* possessed mutations in a low number of basal-like cases (0.6% to 2.9%) and in 0.5% to 1.1% of cases overall in TCGA cohort. The targeted NGS panel of the METABRIC cohort did not include data on mutations in these genes. Amplifications of the three DNA methyltransferases genes were present in 5 of 130 (3.8%) of the claudin-low ER-negative/HER2-negative patients in the METABRIC and in 15 of 143 (10.5%) basal-like patients (Fisher’s exact test *p* = 0.03). Despite the low rates of mutations and amplifications, mRNA over-expression of the three DNA methyltransferases are present in ER-negative/HER2-negative cases, both claudin-low and non-claudin-suppressed, compared to ER-positive/HER2-negative cases (Figure 5). Among DNA methylcytosine dioxygenases that act as demethylases, TET1 is up-regulated in many basal but not claudin-low ER-negative/HER2-negative breast cancers (not shown). These data argue for a role of the post-transcriptional deregulation of DNA methyltransferases in the hypermethylation of ER-negative breast cancers, both with claudin-low and claudin-non-suppressed phenotype.

**Figure 6:**
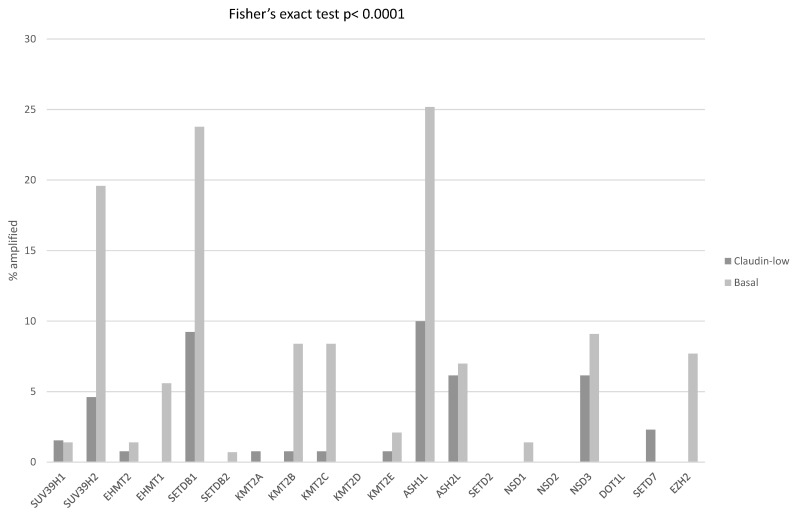
Percentage of amplifications of genes encoding for histone methyltransferases in ER-negative/PR-negative, basal-like breast cancers (grey bars) and ER-negative/PR-negative, claudin-low breast cancers (black bars). Data are from the METABRIC cohort. Fisher’s exact test *p* < 0.0001 for the comparison of the presence of amplifications in any histone methyltransferase in claudin-low versus basal-like breast cancers.

**Figure 7:**
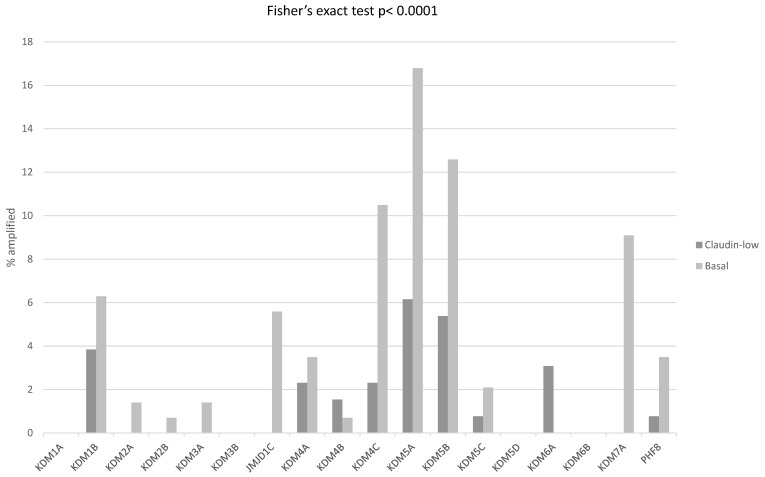
Percentage of amplifications of genes encoding for histone demethylases in ER-negative/PR-negative, basal-like breast cancers (grey bars) and ER-negative/PR-negative, claudin-low breast cancers (black bars). Data are from the METABRIC cohort. Fisher’s exact test *p* < 0.0001 for the comparison of the presence of amplifications in any histone demethylase in claudin-low versus basal-like breast cancers.

Next, enzymes involved in histone methylation and demethylation and histone acetylation and deacetylation were examined for differences between claudin-low and claudin-non-suppressed ER-negative/HER2-negative breast cancers. Histone H3 methyltransferases with mutations in more than 2% of ER-negative/HER2-negative cases included *KMT2C*, *KMT2D* and *SETD2* (8.3%, 7.6% and 3.4%, respectively), with no significant differences between claudin-low and basal-like breast cancers (not shown). In contrast, 64.3% of basal cancers possessed amplifications in one or more H3 methyltransferases, while 34.6% of claudin-low cancers had such amplifications (Fisher’s exact test *p* < 0.0001). Most-frequently amplified methyltransferases included H3K36 methyltransferase *ASH1L* and H3K9 methyltransferase *SETDB1* on chromosome arms 1q22 and 1q21.3, and H3K9 methyltransferase *SUV39H2* on chromosome arm 10p13 (Figure 6). Mutations in histone demethylases were rare in ER-negative/HER2-negative breast cancers but amplifications were common. One or more demethylases were amplified in 53.1% of basal and in 22.3% of claudin-low ER-negative/HER2-negative patients (Fisher’s exact test *p* < 0.0001). Amplified demethylases with the highest prevalence, above 10% in basal cancers, included H3K4 demethylases *KDM5A* from locus 12p13.33 and *KDM5B* from 1q32.1 and H3K9 demethylase *KDM4C* from 9p24.1 (Figure 7).

**Figure 8:**
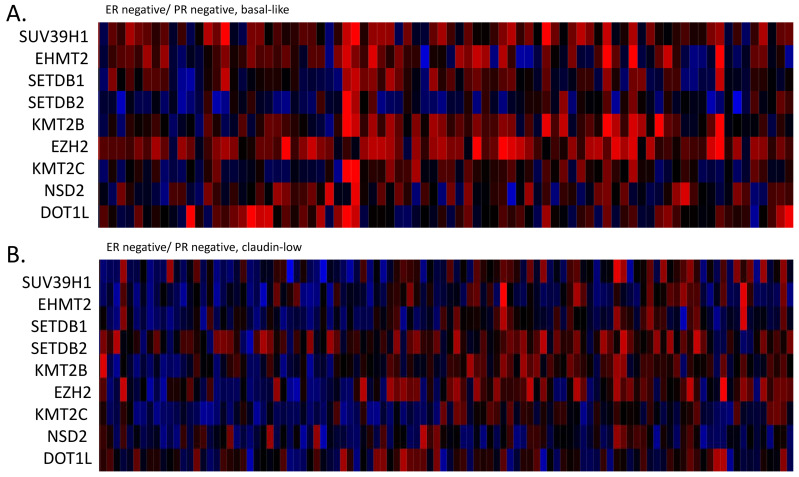
mRNA expression calculated as z-scores relative to all samples (log RNA Seq RPKM) of genes encoding for histone methyltransferases in representative breast cancer cases. (**A**). ER-negative/PR-negative, basal-like cancers, (**B**). ER-negative/PR-negative, claudin-low cancers, Methyltransferases with significantly different expression between the 2 groups are shown. Red color denotes up-regulation and blue denotes suppression.

The mRNA expression of H3 histone methyltransferases was not significantly up-regulated or suppressed in claudin-low breast cancers, with mean z-scores of expression between −0.5 and 0.5 in all. There were statistically significant differences in the mRNA expressions between claudin-low and basal cases, several methyltransferases being moderately up-regulated in the latter (Figure 8). EZH2 methyltransferase, for example, showed a mean mRNA z-score of 1.37 in basal cancers and 0.29 in claudin-low cases (t test corrected for multiple comparisons, *p* = 0.001). mRNA expressions in claudin-low cancers were, for most DNA demethylases, not significantly up-regulated or down-regulated, with mean z-scores of expression between −0.5 and 0.5 except for KDM2B, which was up-regulated (mean z-score 0.87), and KDM4B and KDM5B, which were down-regulated (mean z-scores −1.03 and −0.86, respectively, not shown). The mRNA expression of H3 histone demethylases displayed statistically significant differences between claudin-low and basal cancers in the case of KDM1A (mean mRNA z-score of 1.04 in basal cancers and 0.002 in claudin-low cases, t test corrected for multiple comparisons, *p* = 0.001) and KDM5B (mean mRNA z-score of 0.05 in basal cancers and −0.86 in claudin-low cases, t test corrected for multiple comparisons, *p* = 0.001).

**Figure 9:**
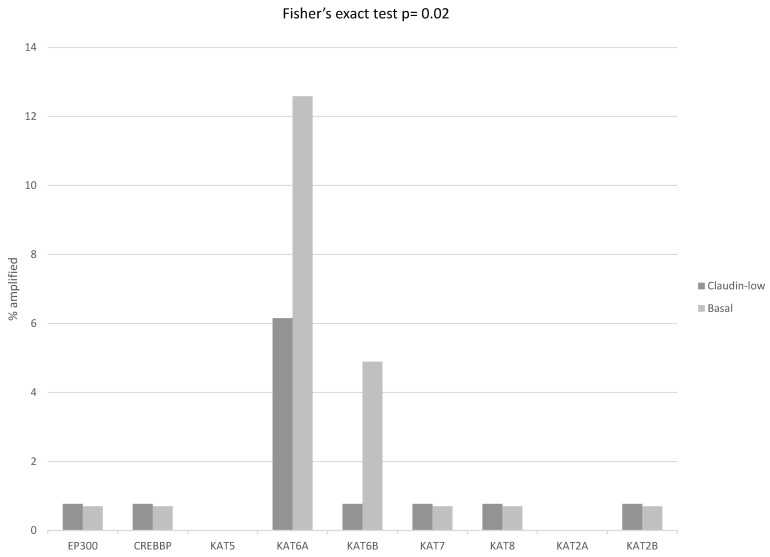
Percentage of amplifications of genes encoding for histone acetyltransferases in ER-negative/PR-negative, basal-like breast cancers (grey bars) and ER-negative/PR-negative, claudin-low breast cancers (black bars). Data are from the METABRIC cohort. Fisher’s exact test *p* = 0.02 for the comparison of the presence of amplifications in any histone acetyltransferase in claudin-low versus basal-like breast cancers.

**Figure 10:**
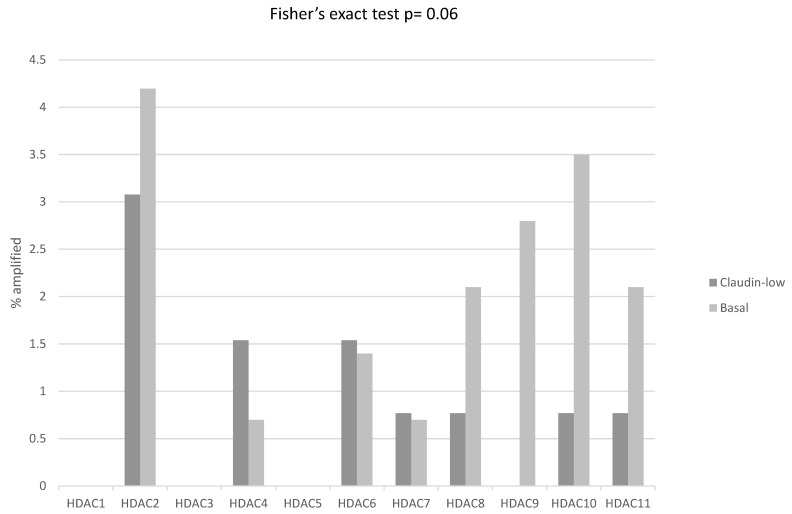
Percentage of amplifications of genes encoding for histone deacetylases in ER-negative/PR-negative, basal-like breast cancers (grey bars) and ER-negative/PR-negative, claudin-low breast cancers (black bars). Data are from the METABRIC cohort. Fisher’s exact test *p* = 0.06 for the comparison of the presence of amplifications in any histone deacetylase in claudin-low versus basal-like breast cancers.

Mutations in histone acetyltransferases were also rare in ER-negative/HER2-negative breast cancers. Amplifications which were more common occurred in 20.2% of basal cancers and 10% of claudin-low cancers (Fisher’s exact test, *p* = 0.02). The higher prevalence of amplifications was in acetyltransferases of the MYST family *KAT6A* and *KAT6B* from chromosomes 8p11.21 and 10q22.2, respectively (Figure 9). Similarly, mutations in histone deacetylases were rare, and amplifications, which were more common, occurred more frequently in basal cancers (16.1% versus 8.5% in claudin-low ER-negative/HER2-negative cancers, Fisher’s exact test, *p* = 0.06, Figure 10). The mRNA expression of histone acetyltransferases in claudin-low cancers displayed no significant up-regulations or down-regulations (mean z-scores of expression between −0.5 and 0.5) except for KAT6B, which was moderately suppressed with a mean z-score of expression of 0.76. No significant differences in the expression of acetyltransferases between claudin-low cases and basal cases were observed (*t* test corrected for multiple comparisons, *p* > 0.05 for all comparisons). The only histone deacetylase with significantly decreased mRNA expression in claudin-low cancers was HDAC11 (mean z-scores of expression −1.23). HDAC11 was also suppressed in basal cancers (mean z-scores of expression −0.93) but the difference in suppression was statistically significant (t test corrected for multiple comparisons, *p* = 0.01).

## 4. Discussion

The claudin-low phenotype was discovered in a subset of breast cancers that are mostly ER-negative/PR-negative and HER2-negative [27]. Claudin-low breast cancers are characterized by the suppressed expression of adhesion proteins and the up-regulation of the EMT program leading to a pro-metastatic phenotype, and producing cells that are able to alternate between epithelial and mesenchymal states. The former have higher proliferation potential and the latter possess motility capabilities favoring movement and tissue infiltration. Previous works have established that, although claudin-low cancers are mostly triple-negative, a smaller sub-set of them can be ER-positive and/or HER2-positive [9,10,28]. Among all cancers with the claudin-low phenotype in the METABRIC cohort, 68.4% are ER-negative/HER2-negative, but 25.3% and 6.3% are ER-positive/HER2-negative and HER2-positive, respectively [9,10]. The EMT core regulators ZEB1, ZEB2, Snail, Slug and TWIST1 are up-regulated in claudin-low cancers independently of the status of ER expression. This is despite the fact that genetic lesions in EMT core regulators, either point mutations or locus amplifications, are not frequent in claudin-low cancers and, interestingly, amplifications of *SNAI2* (encoding for Slug) and *ZEB1* genes are more prevalent in basal triple-negative breast cancers than in claudin-low counterparts. Thus, the up-regulation of the EMT core program is mediated at the transcriptional and post-transcriptional level. A well-established post-transcriptional regulation is through micro-RNA (miR)-mediated suppression, whence mRNAs encoding for core EMT transcription regulators are down-regulated by miRs of the miR-200 family [29]. This miR family encompasses five members, miR-200a, miR-200b, miR-429, miR-200c and miR-141, organized in two chromosome clusters, that are important physiologic regulators of normal mammary glands during the different phases and functions of the mammalian life-time [30]. Thus, the regulations of epithelial and mesenchymal states are physiologically embedded in breast life cycles. miR-200 family members are significantly up-regulated in breast cancer tissues compared with normal breast cancer [31]. miR-200 members are down-regulated in the ER-negative cluster of breast cancer cell lines and up-regulated in ER-positive cell lines, potentially contributing to the differential expression of EMT core regulators [32]. In addition, HER2-positive, triple-negative and luminal B breast cancers show lower expressions of different miR-200 family members compared with luminal A cases [31]. ER-positive breast cancers show higher expressions of miR-200 family members despite the fact that their negative regulator ZEB1 is a transcriptional target of ER [33,34]. This may be explained by the loss of control of ZEB1 expression by ER during EMT [35].

Besides miR modifications, epigenetic activity may regulate EMT core transcription factors without gene defects [17]. The main epigenetic modification of the DNA itself consists of methylations in gene promoters that are facilitated by DNA methyltransferases on cytosines of CpG islands, areas rich in cytosine and guanine dinucleotides frequently found in promoters of methylation-sensitive genes. Examining a panel of established methylation-sensitive genes, it was shown that besides claudin-low breast cancers, basal breast cancers display the down-regulation of these genes. This suggests a role of increased DNA methyltransferase activity associated with the increased expression of human DNA methyltransferases DNMT1, DNMT3A and DNMT3B in several ER-negative breast cancer cases, both claudin-low and basal. On the other hand, these results suggest that DNA methylation is not the sole instigator of EMT core factor expression in claudin-low disease, given that ER-negative cancers without claudin suppression display this feature. Methylated cytosines in DNA serve as docking sites for methylcytosine-binding domain proteins MBD1 to 3 and MeCP2 [36]. Methylcytosine-binding domain proteins then attract histone deacetylases and promote a closed histone configuration associated with gene transcription repression [37]. DNA methylation in triple-negative breast cancers affects many cellular responses, including the response to endoplasmic reticulum stress [38]. In addition, the methylation of homologous recombination gene promoters such as BRCA1 and RAD51C lead to homologous recombination-deficient breast cancer development in sporadic cases [39].

Histone methylation and acetylation is another mechanism of epigenetic gene regulation. The pattern of histone epigenetic enzyme gene alterations is consistent across methylation and acetylation modifiers in ER-negative/HER2-negative breast cancers with rare mutations and more frequent amplifications. Methyltransferases and demethylases show a high prevalence of amplifications in ER-negative/HER2-negative breast cancers and particularly in basal cancers compared to claudin-low counterparts, while acetyltransferases and deacetylases possess a lower prevalence of amplifications but also have a higher prevalence in basal cases. Amplifications of certain H3 methyltransferases, including *ASH1L* and *SETDB1*, show a prevalence of 20% to 25% in basal breast cancers. Similarly, histone demethylases, acetyltransferases and deacetylases display higher mutation rates in basal-like compared to claudin-low breast cancers. The mRNA expression of most of these enzymes is not significantly up-regulated or down-regulated in claudin-low cancers. The only notable exception is HDAC11, which shows a significantly down-regulated mRNA expression. Basal-like cancers also show the down-regulation of HDAC11 but less than claudin-low cancers. Basal cancers also display higher mRNA expressions of several methyltransferases, with the most notable example being H3K27 methyltransferase EZH2, a member of the polycomb repressor complex 2 (PRC2), which mediates gene silencing. Thus, a combination of DNA methylation and the lack of increased histone methylation at H3K27 may define the permissive marks for the claudin-low phenotype. Perturbations to epigenetic regulators and the resulting reversible modifications, at odds with other epithelial mesenchymal regulators, may affect EMT in both directions. For example, a loss of H3K4 methyltransferase KMT2C (also known as MLL3) in breast cancer cells may promote bidirectional movements across the epithelial mesenchymal spectrum depending on the base line state of the cells [40].

The epithelial and mesenchymal states produced by EMT plasticity in cancers are not stable distinct cellular conditions, but rather, intermediate states exist and are common in cancer cells [41]. This fluidity produces cells that can alternate between the two ends of the spectrum, receiving cues from the micro-environment [42,43]. The second messenger adenosine 3′,5′-monophosphate, for example, activates protein kinase A (PKA) in mesenchymal human mammary tumor-initiating cells, which then promotes mesenchymal-to-epithelial transition through epigenetic reprogramming [44]. Reprogramming is mediated by histone demethylase PHF2 (also called KDM7C) in this model and results in cell differentiation and decreased tumor formation. Constitutive PKA activity in mouse models of mammary-gland development led to impaired differentiation and, in mammary tumors, PKA activity promotes cancer cell differentiation [45]. Moreover, the gene encoding for the four regulatory units of the enzyme, PRKAR1A, which negatively regulate the catalytic subunit, is commonly amplified in human breast cancers [45]. In breast cancer stem cells, external signals inducing EMT affect the expression of genes that are initially not expressed but poised for expression, as defined by the presence of both suppressive and activation epigenetic markers in their promoters [46]. Intermediate EMT states that retain characteristics of both epithelial and mesenchymal cells as a result of epigenetic fluidity are more difficult to experimentally ascertain. Increased hydroxymethylcytocine in CpG islands of DNA has been suggested as a marker of intermediate EMT states, arguing for the importance of epigenetic modifications in the process [47].

In conclusion, the claudin-low phenotype in breast cancer is defined by epigenetic states permissive to plasticity allowing cells to undergo changes across the epithelial-to-mesenchymal spectrum. EMP plasticity is associated with stemness, a state of low proliferation and increased drug resistance. Further research will elucidate whether specific epigenetic modifications are absolute prerequisites for the acquisition of EMP and the claudin-low phenotype, with the ultimate goal of devising therapeutic interventions.

## Data Availability

There are no data available beyond those presented in the article.

## References

[1] Giaquinto A.N., Sung H., Miller K.D., Kramer J.L., Newman L.A., Minihan A., Jemal A., Siegel R.L. (2022). Breast Cancer Statistics, 2022. CA Cancer J. Clin..

[2] Sung H., Ferlay J., Siegel R.L., Laversanne M., Soerjomataram I., Jemal A., Bray F. (2021). Global Cancer Statistics 2020: GLOBOCAN Estimates of Incidence and Mortality Worldwide for 36 Cancers in 185 Countries. CA Cancer J. Clin..

[3] Anders C.K., Abramson V., Tan T., Dent R. (2016). The Evolution of Triple-Negative Breast Cancer: From Biology to Novel Therapeutics. Am. Soc. Clin. Oncol. Educ. Book.

[4] Pintens S., Neven P., Drijkoningen M., Van Belle V., Moerman P., Christiaens M.R., Smeets A., Wildiers H., Vanden Bempt I. (2009). Triple negative breast cancer: A study from the point of view of basal CK5/6 and HER-1. J. Clin. Pathol..

[5] Lehmann B.D., Jovanović B., Chen X., Estrada M.V., Johnson K.N., Shyr Y., Moses H.L., Sanders M.E., Pietenpol J.A. (2016). Refinement of Triple-Negative Breast Cancer Molecular Subtypes: Implications for Neoadjuvant Chemotherapy Selection. PLoS ONE.

[6] Burstein M.D., Tsimelzon A., Poage G.M., Covington K.R., Contreras A., Fuqua S.A., Savage M.I., Osborne C.K., Hilsenbeck S.G., Chang J.C. (2015). Comprehensive genomic analysis identifies novel subtypes and targets of triple-negative breast cancer. Clin. Cancer Res..

[7] Asleh K., Riaz N., Nielsen T.O. (2022). Heterogeneity of triple negative breast cancer: Current advances in subtyping and treatment implications. J. Exp. Clin. Cancer Res..

[8] Jiang Y.Z., Ma D., Suo C., Shi J., Xue M., Hu X., Xiao Y., Yu K.D., Liu Y.R., Yu Y. (2019). Genomic and Transcriptomic Landscape of Triple-Negative Breast Cancers: Subtypes and Treatment Strategies. Cancer Cell.

[9] Voutsadakis I.A. (2023). Comparison of Clinical Subtypes of Breast Cancer within the Claudin-Low Molecular Cluster Reveals Distinct Phenotypes. Cancers.

[10] Pereira B., Chin S.F., Rueda O.M., Vollan H.K., Provenzano E., Bardwell H.A., Pugh M., Jones L., Russell R., Sammut S.J. (2016). The somatic mutation profiles of 2,433 breast cancers refines their genomic and transcriptomic landscapes. Nat. Commun..

[11] Sarrió D., Rodriguez-Pinilla S.M., Hardisson D., Cano A., Moreno-Bueno G., Palacios J. (2008). Epithelial-mesenchymal transition in breast cancer relates to the basal-like phenotype. Cancer Res..

[12] Voutsadakis I.A. (2015). The network of pluripotency, epithelial-mesenchymal transition, and prognosis of breast cancer. Breast Cancer.

[13] Brabletz T., Kalluri R., Nieto M.A., Weinberg R.A. (2018). EMT in cancer. Nat. Rev. Cancer.

[14] Mani S.A., Guo W., Liao M.J., Eaton E.N., Ayyanan A., Zhou A.Y., Brooks M., Reinhard F., Zhang C.C., Shipitsin M. (2008). The epithelial-mesenchymal transition generates cells with properties of stem cells. Cell.

[15] Morel A.P., Lièvre M., Thomas C., Hinkal G., Ansieau S., Puisieux A. (2008). Generation of breast cancer stem cells through epithelial-mesenchymal transition. PLoS ONE.

[16] Prat A., Parker J.S., Karginova O., Fan C., Livasy C., Herschkowitz J.I., He X., Perou C.M. (2010). Phenotypic and molecular characterization of the claudin-low intrinsic subtype of breast cancer. Breast Cancer Res..

[17] Tam W.L., Weinberg R.A. (2013). The epigenetics of epithelial-mesenchymal plasticity in cancer. Nat. Med..

[18] Curtis C., Shah S.P., Chin S.F., Turashvili G., Rueda O.M., Dunning M.J., Speed D., Lynch A.G., Samarajiwa S., Yuan Y. (2012). The genomic and transcriptomic architecture of 2000 breast tumours reveals novel subgroups. Nature.

[19] Willett C.G., Chang D.T., Czito B.G., Meyer J., Wo J. (2012). Cancer Genome Atlas Network: Comprehensive molecular portraits of human breast tumours. Nature.

[20] Gao J., Aksoy B.A., Dogrusoz U., Dresdner G., Gross B., Sumer S.O., Sun Y., Jacobsen A., Sinha R., Larsson E. (2013). Integrative analysis of complex cancer genomics and clinical profiles using the cBioPortal. Sci. Signal..

[21] Cerami E., Gao J., Dogrusoz U., Gross B.E., Sumer S.O., Aksoy B.A., Jacobsen A., Byrne C.L., Heuer M.L., Larsson E. (2012). The cBio Cancer Genomics Portal: An open platform for exploring multidimensional cancer genomics data. Cancer Discov..

[22] Perou C.M., Sørlie T., Eisen M.B., van de Rijn M., Jeffrey S.S., Rees C.A., Pollack J.R., Ross D.T., Johnsen H., Akslen L.A. (2000). Molecular portraits of human breast tumours. Nature.

[23] Sørlie T., Perou C.M., Tibshirani R., Aas T., Geisler S., Johnsen H., Hastie T., Eisen M.B., van de Rijn M., Jeffrey S.S. (2001). Gene expression patterns of breast carcinomas distinguish tumor subclasses with clinical implications. Proc. Natl. Acad. Sci. USA.

[24] Herschkowitz J.I., Zhao W., Zhang M., Usary J., Murrow G., Edwards D., Knezevic J., Greene S.B., Darr D., Troester M.A. (2012). Comparative oncogenomics identifies breast tumors enriched in functional tumor-initiating cells. Proc. Natl. Acad. Sci. USA.

[25] Mermel C.H., Schumacher S.E., Hill B., Meyerson M.L., Beroukhim R., Getz G. (2011). GISTIC2.0 facilitates sensitive and confident localization of the targets of focal somatic copy-number alteration in human cancers. Genome Biol..

[26] Li B., Dewey C.N. (2011). RSEM: Accurate transcript quantification from RNA-Seq data with or without a reference genome. BMC Bioinform..

[27] Prat A., Perou C.M. (2011). Deconstructing the molecular portraits of breast cancer. Mol. Oncol..

[28] Fougner C., Bergholtz H., Norum J.H., Sørlie T. (2020). Re-definition of claudin-low as a breast cancer phenotype. Nat. Commun..

[29] Babaei G., Raei N., Toofani Milani A., Gholizadeh-Ghaleh Aziz S., Pourjabbar N., Geravand F. (2021). The emerging role of miR-200 family in metastasis: Focus on EMT, CSCs, angiogenesis, and anoikis. Mol. Biol. Rep..

[30] Roth M.J., Moorehead R.A. (2021). The miR-200 family in normal mammary gland development. BMC Dev. Biol..

[31] Fontana A., Barbano R., Dama E., Pasculli B., Rendina M., Morritti M.G., Melocchi V., Castelvetere M., Valori V.M., Ravaioli S. (2021). Combined analysis of miR-200 family and its significance for breast cancer. Sci. Rep..

[32] Riaz M., van Jaarsveld M.T., Hollestelle A., Prager-van der Smissen W.J., Heine A.A., Boersma A.W., Liu J., Helmijr J., Ozturk B., Smid M. (2013). miRNA expression profiling of 51 human breast cancer cell lines reveals subtype and driver mutation-specific miRNAs. Breast Cancer Res..

[33] Voutsadakis I.A. (2016). Epithelial-Mesenchymal Transition (EMT) and Regulation of EMT Factors by Steroid Nuclear Receptors in Breast Cancer: A Review and in Silico Investigation. J. Clin. Med..

[34] Chamberlain E.M., Sanders M.M. (1999). Identification of the novel player deltaEF1 in estrogen transcriptional cascades. Mol. Cell. Biol..

[35] Spoelstra N.S., Manning N.G., Higashi Y., Darling D., Singh M., Shroyer K.R., Broaddus R.R., Horwitz K.B., Richer J.K. (2006). The transcription factor ZEB1 is aberrantly expressed in aggressive uterine cancers. Cancer Res..

[36] Nickel A., Stadler S.C. (2015). Role of epigenetic mechanisms in epithelial-to-mesenchymal transition of breast cancer cells. Transl. Res..

[37] Jones P.L., Veenstra G.J., Wade P.A., Vermaak D., Kass S.U., Landsberger N., Strouboulis J., Wolffe A.P. (1998). Methylated DNA and MeCP2 recruit histone deacetylase to repress transcription. Nat. Genet..

[38] Ward A.K., Mellor P., Smith S.E., Kendall S., Just N.A., Vizeacoumar F.S., Sarker S., Phillips Z., Alvi R., Saxena A. (2016). Epigenetic silencing of CREB3L1 by DNA methylation is associated with high-grade metastatic breast cancers with poor prognosis and is prevalent in triple negative breast cancers. Breast Cancer Res..

[39] Bonnet E., Haddad V., Quesada S., Baffert K.A., Lardy-Cléaud A., Treilleux I., Pissaloux D., Attignon V., Wang Q., Buisson A. (2022). Alterations in Homologous Recombination-Related Genes and Distinct Platinum Response in Metastatic Triple-Negative Breast Cancers: A Subgroup Analysis of the ProfiLER-01 Trial. J. Pers. Med..

[40] Cui J., Zhang C., Lee J.E., Bartholdy B.A., Yang D., Liu Y., Erler P., Galbo P.M., Hodge D.Q., Huangfu D. (2023). MLL3 loss drives metastasis by promoting a hybrid epithelial-mesenchymal transition state. Nat. Cell Biol..

[41] Voutsadakis I.A. (2019). HER2 in stemness and epithelial-mesenchymal plasticity of breast cancer. Clin. Transl. Oncol..

[42] Cook D.P., Vanderhyden B.C. (2022). Transcriptional census of epithelial-mesenchymal plasticity in cancer. Sci. Adv..

[43] Schwager S.C., Mosier J.A., Padmanabhan R.S., White A., Xing Q., Hapach L.A., Taufalele P.V., Ortiz I., Reinhart-King C.A. (2022). Link between glucose metabolism and epithelial-to-mesenchymal transition drives triple-negative breast cancer migratory heterogeneity. iScience.

[44] Pattabiraman D.R., Bierie B., Kober K.I., Thiru P., Krall J.A., Zill C., Reinhardt F., Tam W.L., Weinberg R.A. (2016). Activation of PKA leads to mesenchymal-to-epithelial transition and loss of tumor-initiating ability. Science.

[45] Ognjenovic N.B., Bagheri M., Mohamed G.A., Xu K., Chen Y., Mohamed Saleem M.A., Brown M.S., Nagaraj S.H., Muller K.E., Gerber S.A. (2020). Limiting Self-Renewal of the Basal Compartment by PKA Activation Induces Differentiation and Alters the Evolution of Mammary Tumors. Dev. Cell.

[46] Hardy K., Wu F., Tu W., Zafar A., Boulding T., McCuaig R., Sutton C.R., Theodoratos A., Rao S. (2016). Identification of chromatin accessibility domains in human breast cancer stem cells. Nucleus.

[47] Lee M.K., Brown M.S., Wilkins O.M., Pattabiraman D.R., Christensen B.C. (2022). Distinct cytosine modification profiles define epithelial-to-mesenchymal cell-state transitions. Epigenomics.

